# Implementation of a high throughput automated platform for residual DNA quantitation

**DOI:** 10.1371/journal.pone.0322133

**Published:** 2025-04-24

**Authors:** Shruti B. Patel, Jon Jurica, Xiaoqing Hua

**Affiliations:** Analytical Research and Development, Merck & Co., Inc., Rahway, New Jersey, United States of America; University of the Punjab Quaid-i-Azam Campus: University of the Punjab, PAKISTAN

## Abstract

Host cell DNA is an impurity from cell-based manufacturing processes that must be controlled and monitored to ensure drug purity and safety. Conventional methods for measurement of residual host cell DNA in therapeutic protein require numerous preparations of plates, DNA extraction from the protein samples, followed by quantification of the extracted DNA using real-time PCR (qPCR). Preparation of plates for extraction is the most laborious step, including numerous manual steps such as sample dilution, standard curve preparation, as well as reagent and sample plate preparation. Additionally, much of the work needs to be performed in a biosafety cabinet to avoid contamination. In this study, a robotic platform using a Gilson liquid handler for plate preparation for rDNA extraction is presented. This automated workflow is not only high throughput, but also shows reproducibility that is equivalent or better as compared to manual workflows. Moreover, this approach is faster than traditional extraction and reduces the risk of human error and variability and eliminates the need for manual pipetting and plate preparation. In this study, automated and manual workflows were performed side-by-side in triplicate from different purification steps from bioreactor to ultrafiltration step. Day to day variability, matrix interference, and spike recovery were assessed to demonstrate the robustness of the automated workflow.

## Introduction

Many therapeutic proteins, such as monoclonal antibodies (mAbs), fusion proteins, bi-specific or tri-specific antibodies are produced using mammalian CHO cells as hosts [1,2]. Host DNA that is not cleared during the purification process and is therefore present in the final product is called residual DNA (rDNA). Host DNA can pose significant health risks and safety concerns. The presence of trace amounts of foreign residual DNA in the body can lead to increased oncogenicity, infectivity, immune rejection, and immunomodulatory risk [3]. Moreover, the host DNA may contain and transfer viral gene which can integrate into the genome and activate oncogenes or inhibit tumor suppressing genes leading to increases risk of cancer [4]. Therefore, it is critical to clear these impurities as much as possible during downstream purification steps. Any remaining rDNA is considered a process impurity that must be measured in the final sample as required by regulatory agencies [5–7]. In fact, maximum allowable limits are set by the World Health Organization (WHO) and the European Union (EU) to ensure the removal of rDNA [8]. In addition to safety reasons outlined above, manufacturers must control the residual DNA as there would be significant financial consequences. If the residual DNA limit was surpassed, the drug would not be suitable to administer into patients and would lead to batch failures potentially costing companies millions [9]. Once residual DNA is detected in the final product, further processing of this material has risks and therefore, must be produced again starting with the full purification process. This would cost companies time, resources and delay the drug to the market. Due to the importance and safety requirements outlined above, sensitive, accurate and rapid methods are needed to support biologics development and ensure clearance of rDNA through downstream purification steps.

Advancements in real time polymerase chain reaction (RT-PCR or quantitative PCR, qPCR) have made it an optimal analytical method for measuring rDNA [10,11] This method allows for the detection and quantitation of low levels of DNA as it can amplify DNA to billions of copies in a short duration (1–2 hours). However, prior to performing RT-PCR, rDNA must be extracted by physical and/or chemical methods to avoid interference from proteins and other matrix with the RT-PCR reaction. Examples of conventional extraction methods of DNA extraction include organic (phenol-chloroform method) [12], non-organic (salting out and proteinase K) [13] adsorption (silica-gel membrane) [14], magnetic beads where DNA binds reversibly to magnetic beads coated with DNA-binding antibody [15], anion exchange technology [16] and density gradients [17].

Commercial kits exist that use magnetic beads to capture and extract rDNA by using a semi-automated high-throughput instrument (such as MagMAX™ Express-96 Deep Well Magnetic Particle Processor) to generate highly pure nucleic acids [18–20]. Despite some automated steps, the approach is still labor intensive with repetitive pipetting and error prone [21,22]. Most importantly, results have shown that performing repetitive pipetting can lead to injury resulting from ergonomic stress [23–25]. In addition to ergonomic concerns, there are numerous reasons why the manual workflows for residual DNA testing have limitations. During process development, the number of samples for residual DNA measurement tends to be high due to the various purification conditions evaluated leading to an analytical bottleneck if performed manually. This leads to performing the maximum number of tests in 96 well plates with many dilutions, reagents mixing, transfer and time sensitive steps. Rapid data turnaround adds a substantial burden on analysts which increases the chance of making pipetting or calculation errors. More importantly, in case of aberrant data, identifying the root cause becomes challenging as it likely was an unintentional error by the analyst. As eluded to earlier, mistakes in a quality control setting would have significant economic consequences. The testing for rDNA also takes place in a clean covered environment which requires practice and patience by the analysts. Lastly, these experimentation take significant amount of an individual’s time and thus, the free time offered via automation can be better utilized in other tasks and responsibilities [26].

Robotic liquid handlers offer a solution to minimize analyst mistakes and prevent the above-mentioned challenges pertaining to sample evaluation hindrances. The devices optimize labor-intensive workflows for large number of samples and eliminate tedious manual sample preparation steps that is a major cause for human error and assay variability. High throughput platforms such as Tecan and/or Hamilton have been adapted to perform different protocols including extraction, qPCR and ELISA [12,13]. However, there are some concerns with these handlers as it pertains to rDNA extraction/preparation such as high cost, large footprint, and proprietary software. In this paper, we utilized the Gilson Liquid handler system as it is affordable, compact and contains a hood/shield (to protect from environmental contamination). In general, automation workflows like the Gilson described in this study provides significant benefit (operational efficiency, product quality, eventual cost savings, safety); however, there are certain barriers that one must consider. The initial investment associated with the procurement of automation and maintenance can be high. The maintenance is a must to ensure accuracy and precision [25]. There is also technology development that must happen to ensure that the assay performs to the same rigor as the manual workflow. Often time, there is troubleshooting and validation activities that must take place before the technology is deployed to support clinical programs. Of course, the automation equipment is specific, so not all assays can be performed on one automation platform.

**Fig. 1:**
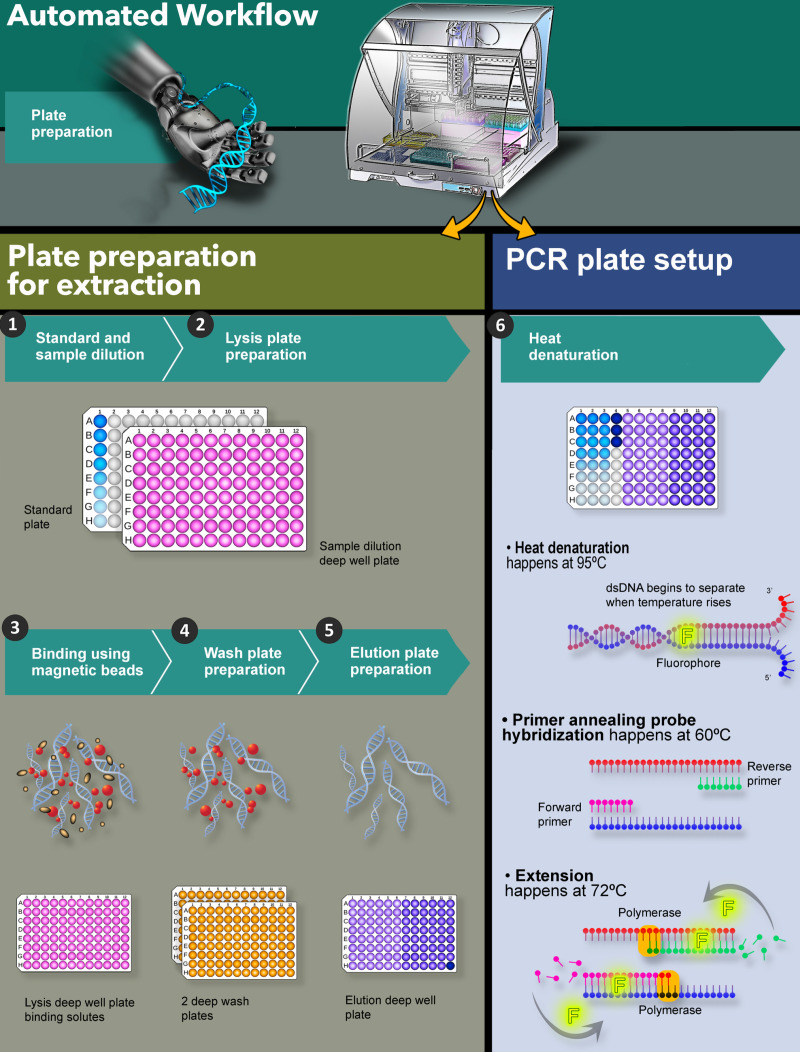
High throughput automated robotic workflow for plate preparation for DNA extraction and DNA quantitation with PCR. Different plate reagents are represented by different colors. For steps 3-5, red dots are magnetic beads, and the brown represents protein and non-DNA impurities.

As illustrated in **Fig 1**, we have utilized the liquid handler to prepare reagents and plates for DNA extraction to maximize the benefits mentioned above. In addition, by combining liquid handler with the MagMAX™, we have created an easy-to-use workflow that is simple, robust, high throughput and accurate. rDNA quantitation across multiple purification steps and various parameters (accuracy, precision, linearity, intermediate precision) are shown below. Most importantly, the automated workflow is equivalent and/or better than the manual workflow and may be implemented for future rDNA quantitation.

## Materials and methods

### Instrumentation

A liquid handling station PIPETMAX®268 (Gilson, United States) equipped with two 8-channel liquid transfer tools (1–20 μL and 20–200 μL) was used for preparation of plates. Extraction was performed on MagMAX™ Express-96 Deep Well Magnetic Particle Processor (Thermo Fisher Scientific, United States). qPCR analyses were performed using an Applied Biosystems 7500 Fast Real-Time PCR System (Thermo Fisher Scientific, United States) equipped with a 96-well heating block.

### Materials

PrepSEQ™ 1-2-3 Nucleic Acid Extraction Kit (Thermo Fisher Scientific, USA), CHO genomic DNA (Cygnus Technologies, USA), TaqMan Universal Master Mix (Applied Biosystems, USA) were used. Primers and probe mix were produced in-house. The CHO DNA concentrate for use as standard was purchased from Cygnus Technologies (Southport, NC, USA). Therapeutic proteins were manufactured in a research setting using CHO cells as hosts. Proteins were purified using mAb purification methods including protein A chromatography, polishing steps and ultrafiltration diafiltration. The experiment was performed for mAb1 using the following process intermediates starting with bioreactor (BR), harvested cell culture filtration (HCCF), Protein A product (PAP), neutralized virus inactivation product (NVIP), filtered neutralized virus inactivation product (FNVIP), polishing chromatography 1 (PC1), polishing chromatography 2 (PC2) and lastly ultrafiltration product (UFP). The concentration of the intermediates varied from ~0.6mg/mL to 52mg/mL. To avoid saturation of the detector, to remain in the range of the calibration curve and to avoid matrix effects - BR and HCCF are diluted 1000-fold and PAP samples are diluted 10-fold.

### Method

The rDNA quantitation method requires three key steps: Sample/plate preparation, DNA extraction and qPCR quantitation (**Fig 1**). Sample preparation (**Table 1**) and the automated workflow using the liquid handler is outlined below. **Table 1** indicates the different plates that must be prepared prior to use in the extraction and qPCR workflow, the purpose of each plate and the components (sample/reagents). The procedure for rDNA extraction was based on manufacturer’s protocol [27]. Proteinase K is added to digest the proteins followed by DNA extraction with magnetic particles. This process is succeeded by a capture, wash and elute sequence to isolate the DNA [27]. The quantitation of rDNA is achieved via qPCR [27,28] Briefly, a PCR master mix is prepared using Taqman Universal Master Mix, primers, probes, and water. Applied Biosystems 7500 Fast Real-Time PCR system is utilized. The data is analyzed using AccuSEQ software version 2.0.

**Table 1.** Plates used during extraction and quantification of residual DNA.

Plate label	Purpose	Components	Procedure
Standard	Generate standard curve for qPCR quantitation. Internal control – spike all samples with 1e5 fg of CHO standard DNA.	CHO DNA reference standard (1e8 fg)	Dilute 1e8 fg standard curve to 10fg with 10-fold serial dilution. 1e5fg is the middle point of the standard curve.
Sample	Sample dilution to ensure rDNA levels within the quantitation range of the standard curve.	Samples, PBS	Instrument is adaptive to samples in both tubes and 96-well plate.
Lysis Plate	Digestion and extraction	A) Digestion: Samples (from sample plate). Spike 1e5 fg (from standard plate), proteinase K enzyme, PBS buffer, 0.5N NaCl. B) Extraction: magnetic beads, lysis and binding solution.	Plate will contain spike samples (contain 1e5fg) and non-spike samples (contain PBS only)
Wash Plate	Remove all non-nucleic acid contamination	Wash buffer	Two wash plate should be prepared
Elution Plate	Elution of purified rDNA	Elution buffer	Pure rDNA will be eluted.
qPCR Plate	Quantitate the amount of rDNA in samples	Standard curve (from standard plate), samples and negative control (from elution plate), primer, probe and water	Placed in Applied Biosystems 7500 qPCR instrument

The lysis, wash and elution plates will be used in the MagMAX™ Express instrument for the digestion and extraction of rDNA. After 30 min of proteinase K digestion, magnetic beads, lysis solution and binding solution are added to the lysis plate for 70 min to extract and elute the rDNA into the elution plate. Lastly, the qPCR plate is prepared by adding the standards (from standard plate), samples and negative controls from elution plate and mixture of primers, probe and water.

### Automation approach

The Gilson liquid handler platform can automate the preparation of all the above plates using the Protocol Builder for PIPETMAX software. Scripts were used and built to provide a customized solution for the analyst to edit detailed parameters such as mixing speed, mixing volume, depth of pipette, aliquot, transfer, type of plate, etc. Trilution 3.0 software is a 21CFR Part11 compliant software that can be used to run the methods (scripts set in the protocol builder). It also contains electronic record management to review and export the data in different file formats. Manual workflow involved pipetting by hand for plate preparation steps.

### Design of experiment

Three replicates of extractions using both automation and manual preparations were performed for process intermediates. Both workflows were subjected to extraction and qPCR quantitation according to the details mentioned above. All the steps in Table 2 were generated using the automated workflow (for the automation approach) except the bulk solution of proteinase K mix, lysis solution and qPCR master mix. The main reason these were prepared separately is they require a large volume and Gilson Pipetmax liquid handler used in this study has a volume limitation (<2 mL). Table 2 only represents one sample and three replicates. This experiment was performed using 8 different samples from process.

**Table 2.** Illustration of experiment design to compare automation approach and manually approach.

Samples number	Automation approach			Manual approach		
	Day 1 replicate	Day 2 replicate	Day 3 replicate	Day 1 replicate	Day 2 replicate	Day 3 Replicate
1	1	1	1	1	1	1
1	2	2	2	2	2	2
1	3	3	3	3	3	3

Spiked samples are created by the addition of a known amount of CHO rDNA (1e5 fg). Spike recovery helps to identify matrix effects and compare the two preparation approaches, i.e., automated workflow vs. manual preparation. The non-spike samples contain PBS instead of rDNA spike. The negative control only contains buffers (no protein) and is designated as the extracted negative control (ENC).

After preparation of the automated and manual plates, automated rDNA extractions were performed with the PrepSEQ^TM^ Residual rDNA Sample Preparation Kit on the MagMAX^TM^ Express 96 magnetic particle processor (Applied Biosystems/Life Technologies, USA). Following that, a qPCR setup was prepared, utilizing serially diluted CHO rDNA. In this step, spike, non-spike, and extracted negative control samples were transferred from the elution plate, which was used in the extraction step, to the qPCR plate. (The extraction plate layout is shown in Supporting information S1 Fig, and the qPCR plate setup is illustrated in Supporting information S2 Fig).

Manual and automation extractions were performed across three different days. From this data, the performance of the automated workflow (in comparison to manual workflow) was assessed, including repeatability (within three plates), intermediate precision (across days), accuracy, linearity, range, and quantitation limit (LOQ).

### Data analysis

Precision was measured with the triplicate rDNA reportable results in PCR wells. Accuracy was determined by measuring the rDNA spike recovery and expressed as %Recovery from the following calculation:


$$Recovery\%=\frac{DNAquantityinspikedsample-DNAquantityinunspikedsample}{Spikequantity}\times 100\%$$

## Results and discussion

In-process (IP) intermediate samples from mAb1 (BR, HCCF, PAP, NIVP, FNVIP, PC1, PC2, UFP) were subjected to both automated and manual workflows for plate preparation, followed by rDNA extraction and qPCR quantitation as described in the methods. Both the manual and automated workflow show the clearance of the rDNA as the protein is processed through the different purification steps. Most importantly, the level of rDNA is comparable for both methods for each process intermediate at the highest and at the LOQ levels.

### Accuracy/Precision/Intermediate precision

The accuracy of the method was assessed by calculating the rDNA spike recovery of the eight in-process samples by the calculation in the methods. The acceptable range of spike recovery set in the current method is between 50% and 150%.

**Fig. 2:**
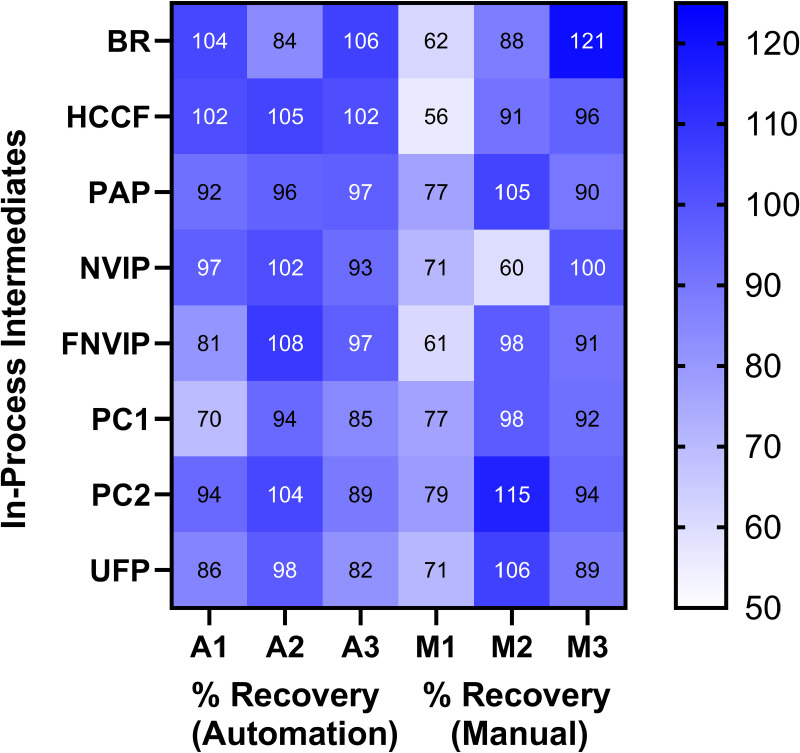
Accuracy of automation and manual workflow for mAb1. A heat map of the various intermediates is compared with automation and manual workflow (n =3) with lighter and darker blue shades indicating varying % recovery. The % recovery values are indicated by the *number.*

**Fig. 2** shows the accuracy results of the eight IP samples spiked with 1e5 (100,000) fg CHO rDNA from the standard curve. All % recoveries were within the 50% to 150% criterion of the method. The automation has 70% to 108% and manually pipetting has 56% to 121% demonstrating that the automation method can accurately measure CHO rDNA in mAb1.

**Fig 3:**
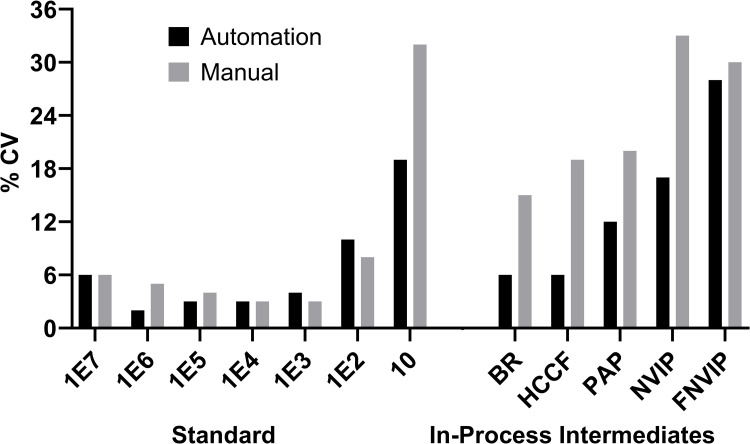
Intermediate precision for automated and manual experiments for standards and in process intermediates. The % CV of standard and in process intermediates is shown for both automated and manual workflow across 3 different days.

The precision of each in-process sample for each day (run performed in triplicate) is shown in **Fig 3**. The variability of each sample for the automation workflow is consistent and within 10% for most samples/days. However, the manual workflow has more variability in terms of rDNA per triplicate sample performed on the same day. The FNVIP in-process intermediate shows the highest variability for both workflows mainly due to the levels of rDNA being close to LOQ. Overall, the calculated % CV for automated workflow is not only comparable but much more consistent than manual workflows. The higher rDNA containing in-process intermediates BR, HCCF, PAP, and NVIP shows a % CV of < 17% for automation workflow and the lowest %CV for manually workflow starts from >15%. For overall day to day variability, NVIP has variability more than 30% in manually run, but each individual run has variability within 30% of range. However, automation has variability less than 30% for both individual and day to day. In addition to differences within each day, the variability of the workflows was assessed across three days (n=9 for each in-process sample for each workflow). As eight in-process samples were compared, this resulted in a total of 72 extractions/workflow. In general, the %CV across 3 days is much more consistent for the automation workflow than the manual. The graphically data for the automation and manual workflow are shown in **Fig 3**. For the earlier steps of the mAb1 process (BR, HCCF), the rDNA is very high as they require a 1000x dilution for values to fall in the linear range. The %CV for these samples is ~6% for the automation workflow while it is slightly higher ~15% for the manual workflow. PAP step as expected clears significant amount of rDNA as it purifies mainly mAb1 away from other proteins. NVIP step is a viral inactivation step that has little impact on the clearance of rDNA. Therefore, the PAP and NVIP show similar levels of rDNA in both workflows. Again, the % CV is higher in the manual workflow for PAP (20% vs 12%) and NVIP (33% vs 17%). These values are the most critical that ensure the process is clearing the rDNA, and at the same time having enough rDNA to accurately quantitate the amount. Lastly, the in-process samples FNVIP has low levels of rDNA (**Fig 5).** One should not over analyze % CV values for this sample as they contain extremely low quantities of rDNA thus slight variation in the repeatability is expected. For PC1, PC2, and UFP samples the rDNA levels are below the LOQ levels (Supporting information l S3 Table and S4 Table) for the rDNA assay (< 10fg), thus the % CV for these samples is not calculated nor and shown in **Fig 3**.

**Fig 4:**
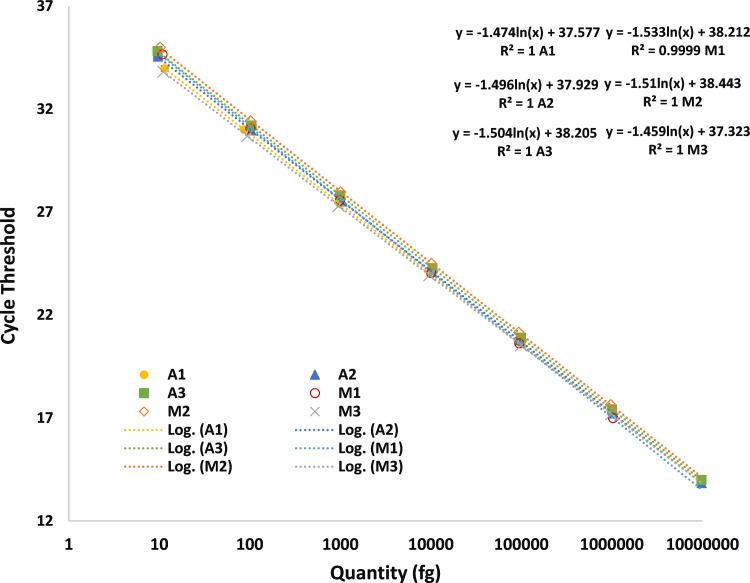
Determination of linearity for automatic and manual workflow Standards were run in triplicate using both workflows (A -automated, M- manual) over three days. Linearity was fit logarithmically and the slope, intercept and R^2^ value calculated.

**Fig 5:**
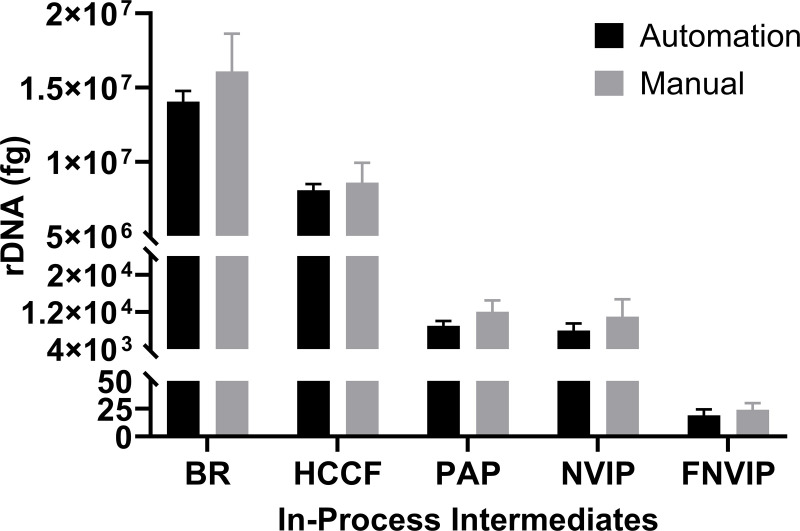
Amount of rDNA present in various in process intermediates by automation and manual workflow.

### Linearity

Linearity was reported as the linear relationship between the mean measured (observed) CHO rDNA quantity (fg) and the theoretical (expected) value in the spiked samples. The two acceptance criteria for linearity as outlined are 1) The mean observed CHO rDNA quantity (fg) in the test samples must have a linear relationship to the theoretical (fg) in plot 2) The coefficient of determination (R^2^) must be ≥0.95.

**Fig 4** shows that the linear range of the method for the quantitation of CHO rDNA in standard is from 1e1 fg to 1e7 fg of rDNA with an R^2^ = 1. The y-intercept = ~ -1.5 the slope = 1.

Linearity between manual preparation (M1, M2, M3) and automation approach (A1, A2, A3) are comparable, as indicated in **Fig 4**. Although the linearity is comparable between both workflows, the linearity from automation is tighter or more consistent than manual preparation from day-to-day method performance as indicated by the variability in the slopes.

### Range

The range for the method was reported as the highest to the lowest level that passes the acceptance criteria for accuracy, precision and linearity. Based on the linearity, accuracy and precision data from this study, the method for the quantitation of CHO rDNA standards are linear, accurate and precise over the range of 1e1 fg to 1e7 fg. **Fig 5** details the amount of rDNA present at various in process intermediate steps. This data set is critical information for process development that ensures clearance of rDNA. In the event, that there is still significant residual DNA present, process must be optimized to have a final produce devoid of rDNA. The residual DNA levels in **Fig 5** are typical of mAb purification. The most DNA is present in the bioreactor samples as they contain the host (CHO cells) which produced the residual DNA. HCCF samples are cell free samples, which typically pass through a membrane filter removing the host cells from the product. This reduces some rDNA levels however, since no chromatography is performed at this step, the levels remain elevated. During affinity purification using protein A, the antibody is captured by the column and most residual DNA flows through. This results in a significant decrease in rDNA (1e7 fg to 1e4 fg). NVIP is a low pH hold step which only precipitates host cell proteins and as expected does not lead to any DNA clearance. FNVIP is a filtration step which uses charged and/or hydrophobic depth filters to assist in clearance of residual DNA and residual host cell proteins. Finally, ion exchange chromatography uses charged beads to ensures residual DNA are well below the limits [27].

The comparison of both automated and manual workflow shows comparable values across the various intermediate steps and is discussed below. For HCCF samples, the automated workflow measured an average of 8079079 fg while the manual workflow measured an average of 8592923 fg. The average quantity of residual DNA from manual workflow is slightly higher than the automation workflow for some samples like BR, HCCF and NVIP samples due to the little higher quantity detection on day 2 and PAP day 3 has little higher value compare to other two days. Upon further processing of therapeutic protein (PC1 onward), the levels or rDNA are below the limit of quantitation for both manual and automation workflows (Supporting information S3 Table and S4 Table).

### Discussion

Having a high throughput, easy to use, automated method is critical to increase the speed of drug development for biopharmaceutical manufacturers. Moreover, accurate and sensitive measurement of the product or in-process impurity is of importance for process development. The results presented in this report prove that liquid handler robot removes the physical strain and human error of plate preparation required for the rDNA assay. As numerous plates are prepared and dilutions are made prior to the rDNA extraction, it is not uncommon to have errors. Here, it was demonstrated that automation workflow is comparable or better than the manual workflow. The range for the automation workflow is also comparable for both dirtier samples containing significant rDNA in the earlier purification steps (HCCF) as well as drug substance with minimal impurity. Parameters such as linearity, accuracy, intermediate precision were evaluated, and results are very positive. We have implemented this automated workflow as part of our everyday operations to test process samples. The manual workflow can create constraints such as backlog of samples and having timely data allows for further optimization of purification steps and progress process development faster.

Systems that have performed similar automated residual DNA workflow include QIAsymphony and Tecan Evo Freedom EVO 200 [29]. These are highly sophisticated instruments that have additional ability including interfacing with other equipment/detectors via robotic arms and customizable number of heads between 1–384 channels [26]. Some of these such as QIAsymphony are also specific for RNA, DNA and protein purification and require significant effort to configure to a different workflow. Compared to these liquid handling platform, Gilson Pipetmax is classified at a much lower tier form as a pipette assisting robot, with a significantly lesser cost and smaller footprint [30]. Of course, Gilson Pipetmax has its limitation such as no robotic arms and usually one head with 1–8 channels. It is this simplicity that we prefer as the other rDNA steps are the same (rDNA Extraction and qPCR). The Gilson Pipetmax required least change from the manual workflow while alleviating the bottleneck of testing, analyst time, minimization of errors. Lastly, the versatility of the Gilson Pipetmax has been showcased in the literature for other studies such as kinase profiling [31], virus study monitoring and gene expression study of parkinson disease [32].

### Conclusion

Here, we demonstrated a facile automation approach using Gilson platform for rDNA sample preparation, with better accuracy, precision, and intermediate precision in comparison to manual preparation. The linearity and range are comparable to manual preparation. The automation approach is high-throughput in nature, the application of which can be expanded to not only supporting process development and characterization, but also leveraged to support process scale up, transfer, modeling, etc. The scripts for operating the liquid handler can be locked in application only, therefore the automation approach has great potential to be used in a QC space for release purposes as well.

## Supporting information

S1 Fig “Extraction (Lysis) Plate Layout for Automation and Manual Experiments. **”** The plate layout for extraction is shown in S1 Fig. The layout is in the form of a 96 well plate with non-spiked and spiked samples run in triplicate (occupying 3 wells).
(TIFF)

S2 Fig “PCR Plate Layout for Automation and Manual Experiments”. S2 Fig illustrates the 96-well plate layout for the quantitative PCR (qPCR) analysis. Columns 1, 2, and 3 contain serially diluted standards, ranging from 1e7 fg to 1e1 fg. Columns 5, 6, and 7 are designated for non-spiked samples sourced from the elution plate, while columns 9, 10, and 11 contain spiked samples also obtained from the elution plate. Each sample type was prepared in triplicate reactions.
(TIFF)

S3 Table “Accuracy and precision data of the automation workflow for in-process samples. **”**S3 Table shows the amount of rDNA over 3 different days performed by automation for various in-process samples.
(TIFF)

S4 Table “Accuracy and precision data of manual workflow for In-process samples. **”**S4 Table shows the amount of rDNA over 3 different days performed by manual for various in-process samples.
(TIFF)

## Abbreviations

mAbs: Monoclonal antibodies
CHO: Chinese Hamster Ovary cells
rDNA: residual deoxyribonucleic acid
WHO: World Health Organization
EU: European Union
RT-PCR or quantitative PCR, qPCR: Polymerase chain reaction
BR: Bioreactor
HCCF: Harvested cell culture filtration
PAP: Protein A product
NVIP: Neutralized virus inactivation product
FNVIP: Filtered neutralized virus inactivation product
PC1: Polishing chromatography 1
PC2: Polishing chromatography 2
UFP: Ultrafiltration product
ENC: Extracted negative control
LOQ: Limit of quantitation
IP: In-process
% CV: Coefficient of Variation
M: Manual preparation
A: Automation approach
fg: Femtogram
µL: Microliter
