# Supplementary figures and images for: Implementation of a high throughput automated platform for residual DNA quantitation
Source: PLoS One. 2025 Apr 24;20(4):e0322133. doi: 10.1371/journal.pone.0322133 (PMC12021156; doi:10.1371/journal.pone.0322133)

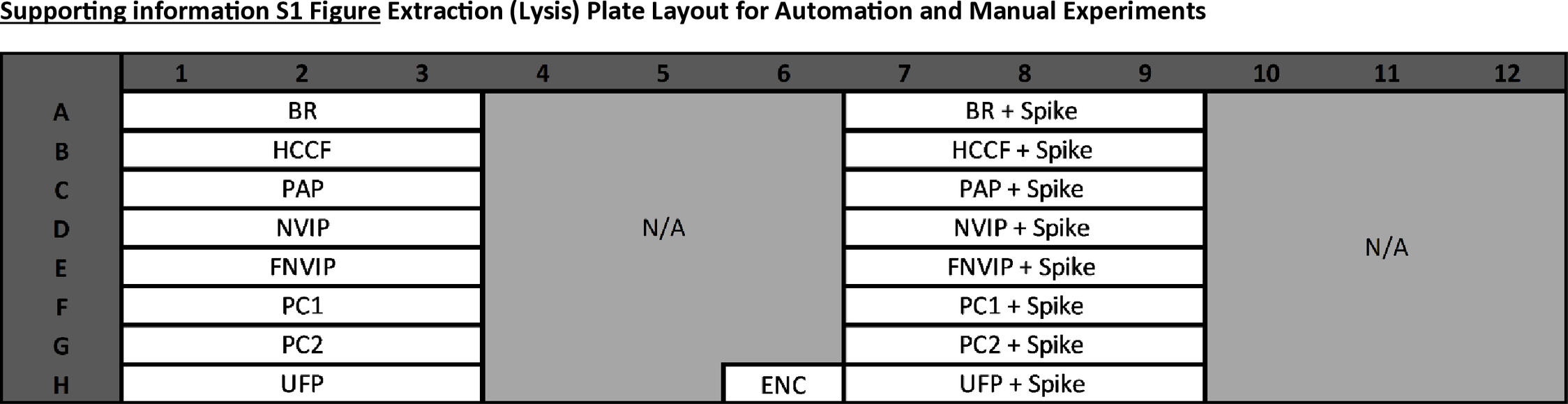

Supplement: S1 Fig — ” The plate layout for extraction is shown in S1 Fig. The layout is in the form of a 96 well plate with non-spiked and spiked samples run in triplicate (occupying 3 wells). (TIFF) [file pone.0322133.s001.tif]

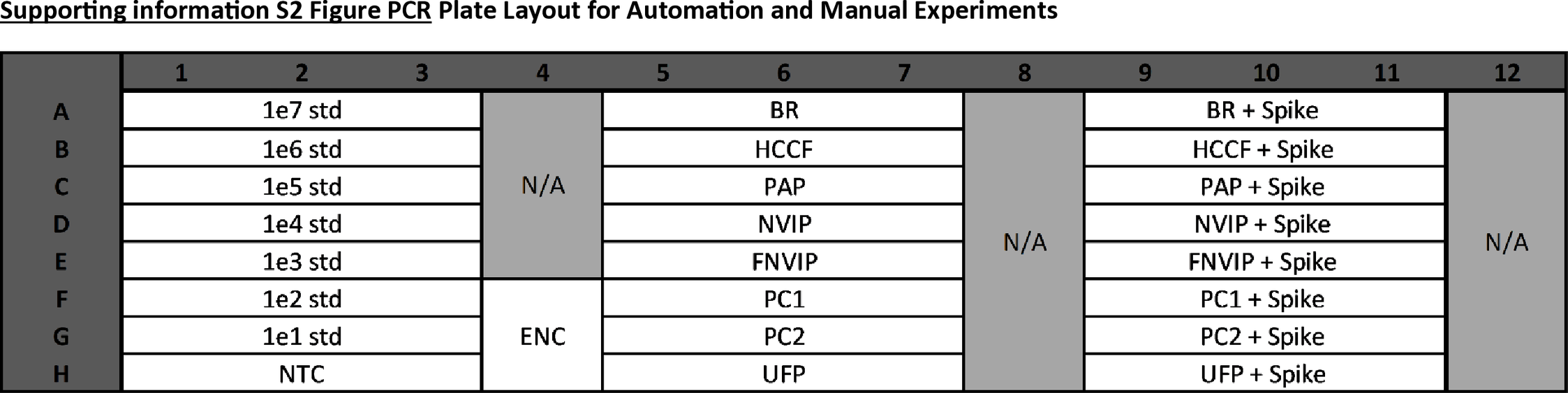

Supplement: S2 Fig — illustrates the 96-well plate layout for the quantitative PCR (qPCR) analysis. Columns 1, 2, and 3 contain serially diluted standards, ranging from 1e7 fg to 1e1 fg. Columns 5, 6, and 7 are designated for non-spiked samples sourced from the elution plate, while columns 9, 10, and 11 contain spiked samples also obtained from the elution plate. Each sample type was prepared in triplicate reactions. (TIFF) [file pone.0322133.s002.tif]

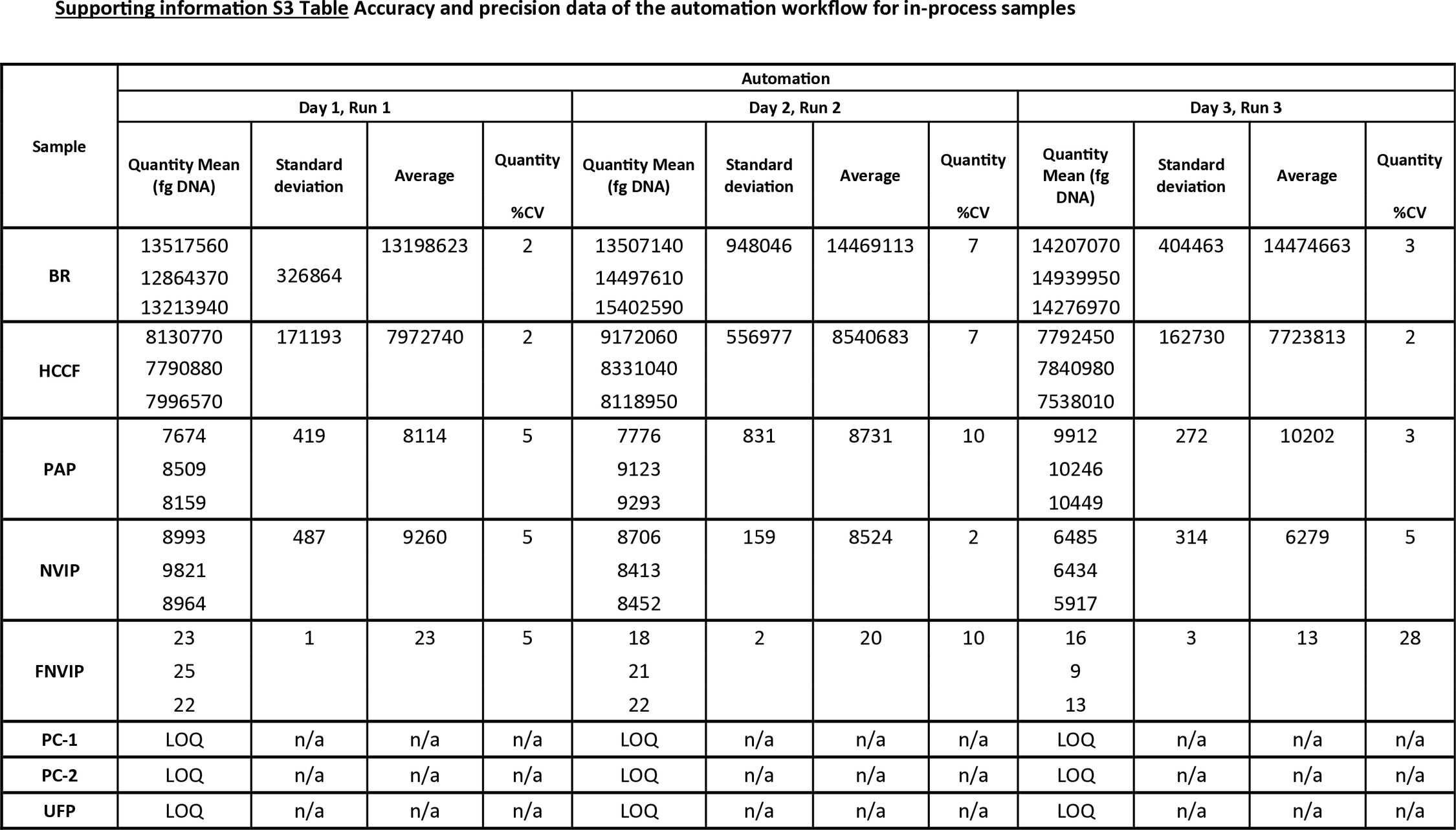

Supplement: S3 Table — ”S3 Table shows the amount of rDNA over 3 different days performed by automation for various in-process samples. (TIFF) [file pone.0322133.s003.tif]

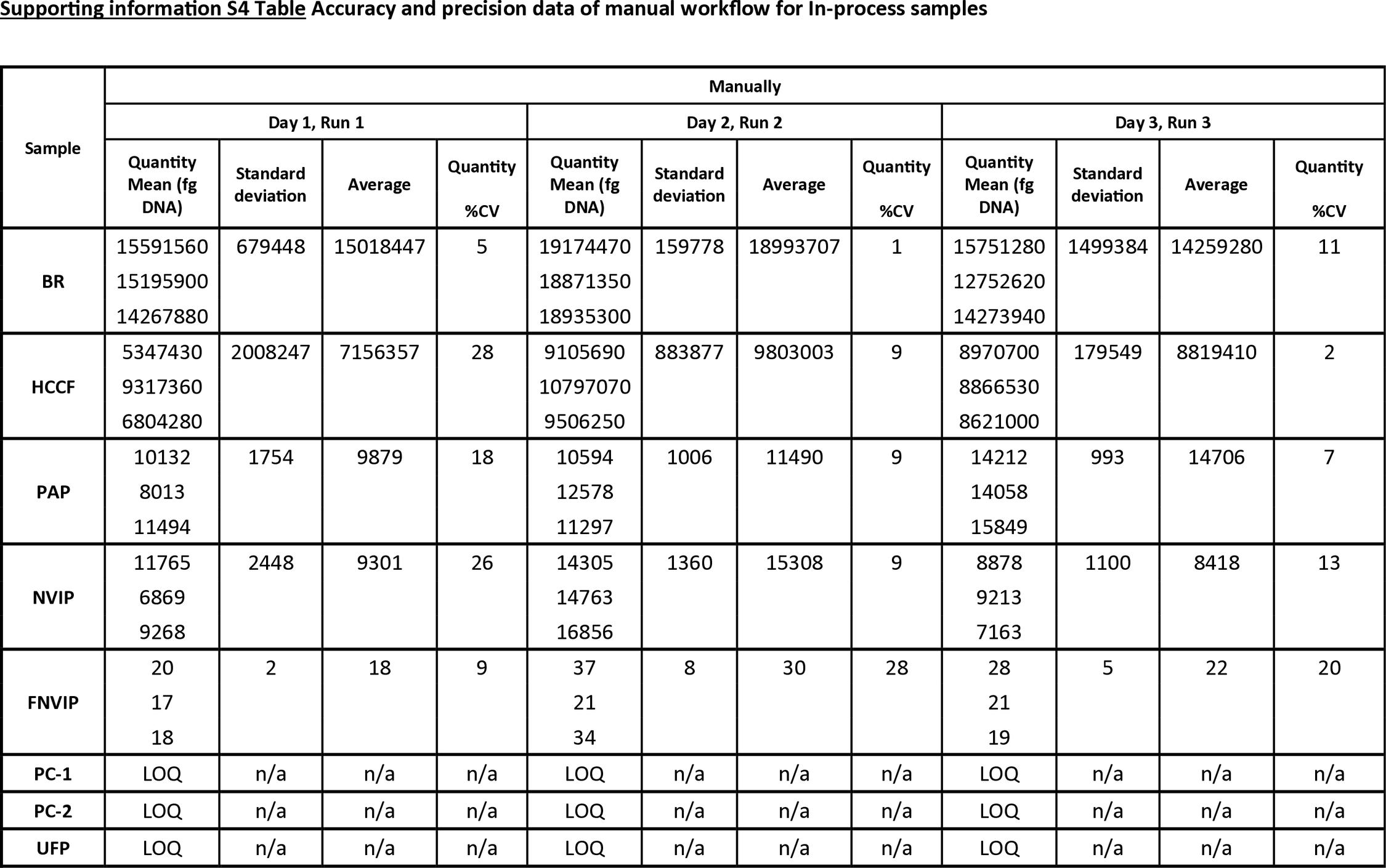

Supplement: S4 Table — ”S4 Table shows the amount of rDNA over 3 different days performed by manual for various in-process samples. (TIFF) [file pone.0322133.s004.tif]
